# Arthroscopic osteochondral autologous transplantation for the treatment of osteochondritis dissecans of the femoral head

**DOI:** 10.1051/sicotj/2016044

**Published:** 2017-03-06

**Authors:** Soshi Uchida, Hajime Utsunomiya, Eisaburo Honda, Shiho Kanezaki, Eiichiro Nakamura, Cecilia Pascual-Garrido, Akinori Sakai

**Affiliations:** 1 Wakamatsu hospital of University of Occupational and Environmental Health Kitakyushu, Fukuoka Japan

**Keywords:** Hip arthroscopy, osteochondral autograft transplantation, osteochondritis dissecans, femoral head

## Abstract

Osteochondritis dissecans (OCD) of the femoral head is an unusual cause of hip pain. It can be associated with other intra-articular pathologies including: acetabular labral tears or bone deformities such as Legg-Calve-Perthes Disease (LCPD). In this article, we propose a modern surgical technique using an arthroscopic antegrade and retrograde osteochondral autologous transplantation (OAT) procedure for assessing and treating OCD lesions of the femoral head.

## Introduction

Osteochondritis dissecans (OCD) in the hip is an uncommon condition characterized by the separation of the osteochondral lesion from the subchondral bone. Lindholm et al. reported 36 patients treated for OCD lesions of the elbow, ankle, and hip during a period of 20 years. Only six (five men and one woman) of the 36 patients had OCD lesions of the hip [1]. Several studies have shown a higher prevalence of OCD of the femoral head in patients with Legg-Calve-Perthes disease (LCPD) [2–4].

The vast majority of patients with symptomatic OCD lesions of the femoral head require surgical management when nonsurgical treatment fails. Fragment removal can cause joint incongruity predisposing toward osteoarthritis. Thus ideally, the aim of surgical interventions should be to restore the articular surface congruity and preserve normal joint kinematics. Numerous surgical procedures including: fragment fixation with bone pegs, osteochondral autograft transplantation (OAT), fresh OAT allograft, and joint arthroplasty have all been reported. Arthroscopic osteochondral autologous transplantation (OAT) is a well-developed and promising technique for the treatment of isolated, full-thickness cartilage lesions in knee and ankle joints [5–7]. In the literature, however, most of the patients treated for osteochondral lesions in the hip require an open surgical dislocation procedure.

Hip arthroscopy is a fascinating tool for assessing and treating hip pathologies including labral tears and femoroacetabular impingement (FAI). Our previous report demonstrated antegrade OAT for treating OCD lesion of femoral head as an innovative and novel technique [8]. In addition, retrograde OAT for chondral lesion of the medial central area of the femoral head has also been reported [9]. The purpose of this article was to demonstrate a novel surgical technique and case presentation of arthroscopic OAT for the treatment of OCD lesion of femoral head.

## Surgical technique

### Hip arthroscopy

The patient was placed in a modified supine position on the traction table (Hip Positioning System, Smith & Nephew Endoscopy, Andover, MA), under general and epidural anesthesia, with a well-padded perineal post. Countertraction on the contralateral leg was obtained by abduction. The involved hip was internally rotated and slightly flexed.

First, arthroscopic evaluation for intra-articular lesions, including labral tearing and associated cartilage damage, was performed through an anterolateral portal (ALP) and a midanterior portal (MAP). Intra-portal capsulotomy using a beaver knife (Becton Dickinson, Franklin Lakes, NJ) was completed to improve accessibility of the scope and surgical instruments.

### Case 1: antegrade osteochondral autograft

**Figure 1.**
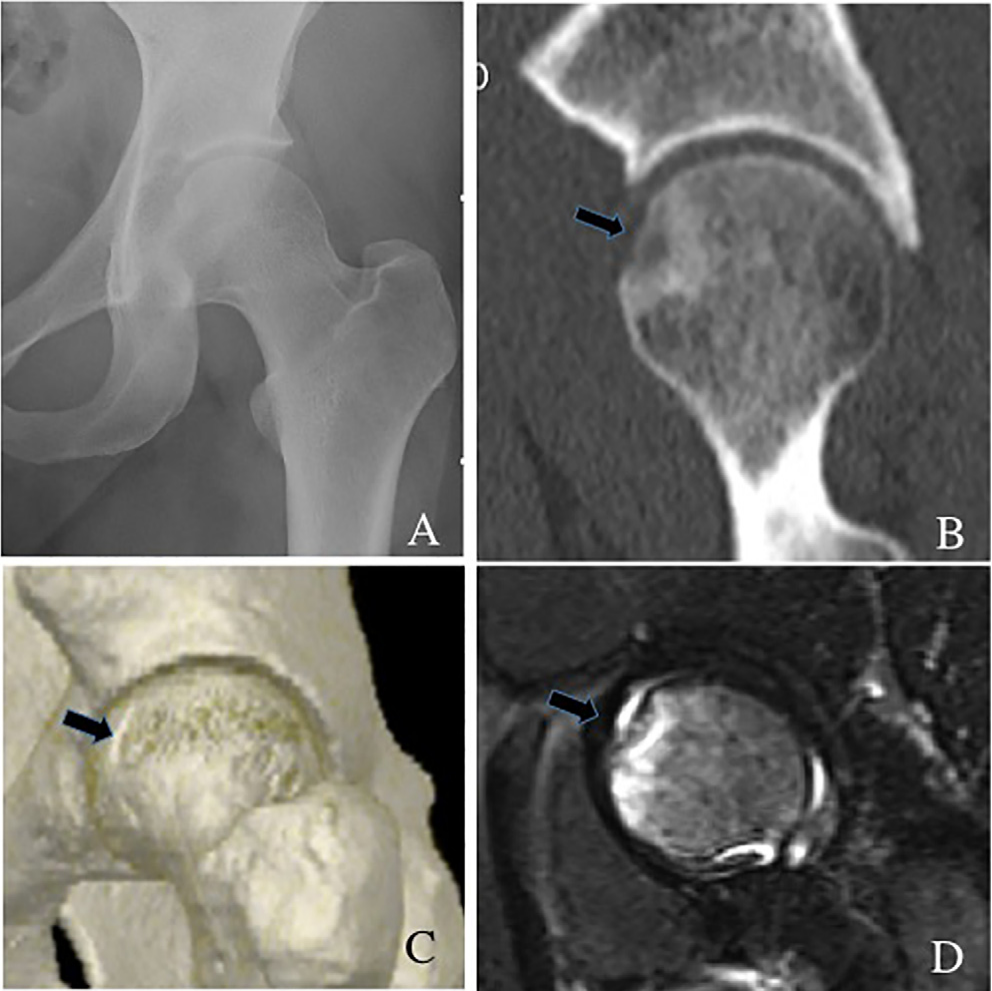
(A) A round radiolucent lesion (arrow) suggests an osteochondritis dissecans (OCD) lesion at the anterosuperior portion of the left femoral head. (B) Preoperative oblique sagittal view of computed tomography showing a round radiolucent lesion at the anterosuperior site of the femoral head. (C) 3-dimensional computed tomography also showing an OCD lesion (arrow) at the anterosuperior site of the femoral head. (D) Coronal magnetic resonance imaging (MRI) view (short inversion time inversion-recovery sequence) showing a high-intensity round lesion (arrow) at the anterosuperior portion of the femoral head.

**Figure 2.**
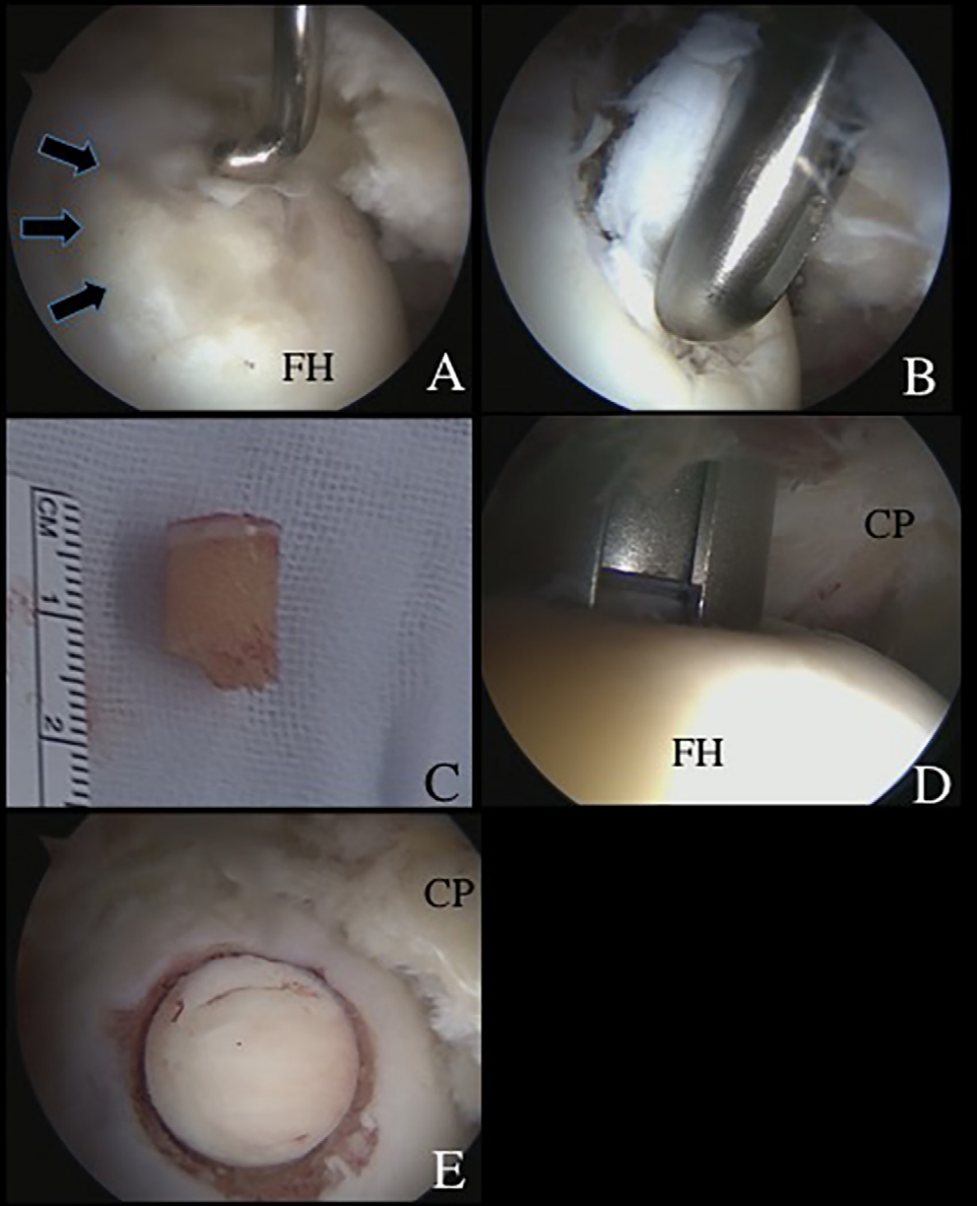
Surgical findings and technique. (A) An osteochondritis dissecans (OCD) lesion (arrows), classified as International Cartilage Repair Society grade III, was observed at the anterosuperior femoral head, with the arthroscope viewing from the anterolateral portal (ALP). A probe through the proximal midanterior portal (PMAP) was used to evaluate the OCD lesion. (B) The degenerative OCD lesion was resected under direct visualization from the ALP. (C) A cylindrical autologous osteochondral graft (8.5 mm in diameter) was harvested arthroscopically from the ipsilateral knee joint. (D) A drill guide was introduced through the PMAP, viewing from the ALP. The subchondral bone was drilled to a depth of 14 mm. The dilator was inserted into the drill guide and tapped to the desired depth. (E) The autologous osteochondral graft was tamped into the lesion until the articular surface was flush with the host joint surface. (CP: capsule; FH: femoral head.)

A 40-year-old female patient presented to us with a 12-month history of left hip pain. Physical examination showed limited range of motion (ROM) with a flexion of 90° and internal rotation of 15°. She had positive impingement sign and hip dial test. Plain radiographs showed a lateral center edge angle (LCEA) of 23° and an alpha angle of 65° suggesting borderline hip dysplasia and concomitant cam impingement (Figure 1A). Computed tomography (CT) and magnetic resonance imaging (MRI) confirmed an OCD lesion of the femoral head (Figures 1B and 1C). Partial tearing at the anterior site of the acetabular labrum (9:30 position) and associated cartilage softening at rim lesion were observed. This last was classified as grade 1 of the Multicenter Arthroscopy of the Hip Outcomes Research Network (MAHORN) [10]. The thermoplasty was carried out using a radiofrequency probe for the treatment of the partial tearing of the labrum (VAPER, DePuy Mitek, Raynham, MA). Next, the OCD lesion was observed at the anterosuperior portion of the femoral head (Figure 2A). A proximal midanterior portal (PMAP) was established to access the OCD lesion. This was performed with the hip at 10° of extension. The condition of the cartilage was evaluated using a probe and determined that refixation was not appropriate (Figure 2A). A decision was made to proceed with osteochondral transplantation. Next, viewing from the ALP, the degenerative cartilage lesion was resected using a shaver (DYONICS BONECUTTER^TM^ PLATINUM 4.5 mm, Smith & Nephew Endoscopy, Andover, MA) and a curette inserted through the PMAP (Relevator, DePuy Mitek, Tokyo, Japan) (Figure 2B). The OAT technique was performed using the MosaicPlasty autologous osteochondral grafting system (Smith & Nephew Endoscopy, Andover, MA). The size of the lesion was determined to be 8.5 mm in diameter. This was assessed making sure the chisel was perpendicular to the articular surface (Smith & Nephew Endoscopy, Andover, MA). After releasing traction a cylindrical autologous osteochondral graft (8.5 mm diameter) was harvested arthroscopically from the ipsilateral knee joint with full extension of hip and knee (Figure 2C). The superior lateral aspect of the intercondylar notch was used as the donor site. The Chizel (Smith & Nephew Endoscopy, Andover, MA) was introduced through the superolateral portal in the knee and driven into the bone to a depth of 12 mm. The harvester, containing the graft, was then removed by twisting the T-handle.

At the recipient site, a drill guide at a 90° angle to the recipient site was inserted, through the PMAP, while viewing through the scope located in the ALP. The subchondral bone was drilled to a depth of 14 mm. The dilator was inserted into the drill guide and tapped to the desired depth. The osteochondral autograft was placed over the lesion and tapped into position, until articular surface, making sure it flushed with the host joint surface (Figures 2D and 2E).

After the central compartment procedure was finalized, the traction was released to assess the peripheral compartment. A cam lesion that had diffuse cartilage damage (International Cartilage Research Society (ICRS) grade II) was seen at the femoral head-neck junction. Arthroscopic dynamic assessment confirmed cam impingement. Thus, cam osteoplasty was carried out using a motorized round burr. Lastly, capsular closure was performed in the hip at 40° of flexion, through the MAP as described previously. A total of two side-to-side stitches were placed to close the capsule. Modified Harris hip score (MHHS) improved from 59.4 preoperatively to 88 at three years after surgery. Non-arthritis hip score improved from 72.5 to 87.5 at three years after surgery.

### Case 2: retrograde osteochondral autograft transplantation

**Figure 3.**
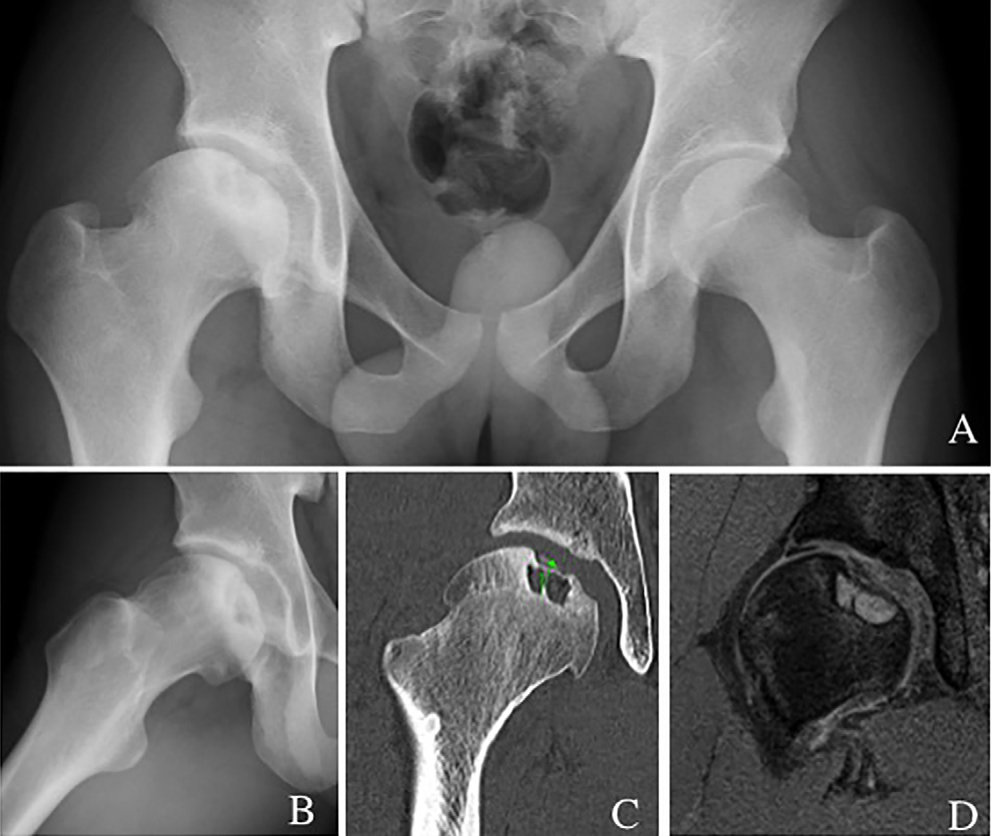
(A and B) Preoperative anteroposterior radiograph and modified Dunn view (45° flexion and 20° external rotation) showing OCD lesion of the aspherical shaped femoral head. (C) CT showing OCD lesion including subchondral cystic lesion of the femoral head. (D) MRI showing OCD lesion and subchondral cystic change.

A 18-year-old badminton male player presented with right hip pain for the past 18 months. The pain was worsened by walking and stepping. Physical examination showed pain on internal rotation and hip flexion at 90°. Plain X-ray identified a lateral center edge angle (LCEA) of 18° and an OCD lesion with concomitant deformity of the femoral head. OCD lesion of the femoral head, hip dysplasia, and Perthes like deformity of proximal femur was diagnosed (Figures 3A and 3B). Perthes like deformity was classified as Stulberg grade I. CT and MRI demonstrated an OCD lesion of femoral head partially separated from the subchondral bone in association with a cystic lesion in the subchondral bone at the central-superior portion of the femoral head [11].

**Figure 4.**
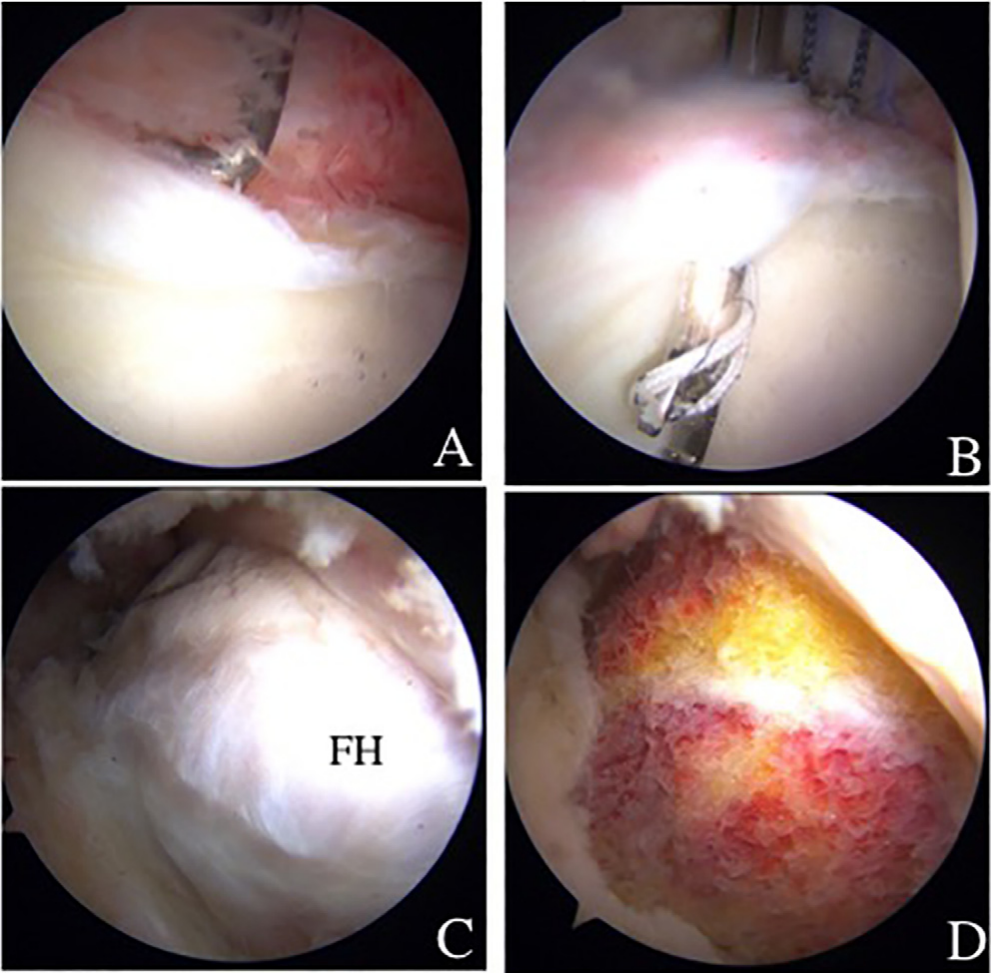
(A) Arthroscopic findings from anterolateral portal showing anterosuperior labral tear. (B) Acetabular labral repair with suture anchor fixation. (C) Arthroscopic findings from midanterior portal showing cam impingement. (D) Osteoplasty was performed.

**Figure 5.**
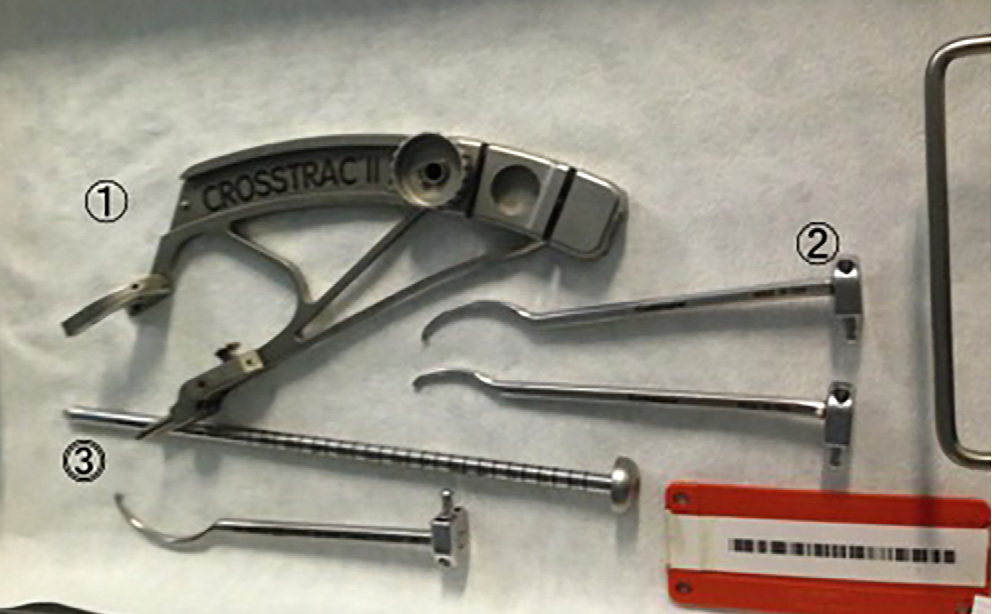
A CROSSTRAC II drill guide system. (1) Drill guide, (2) aimer, (3) drill tube.

**Figure 6.**
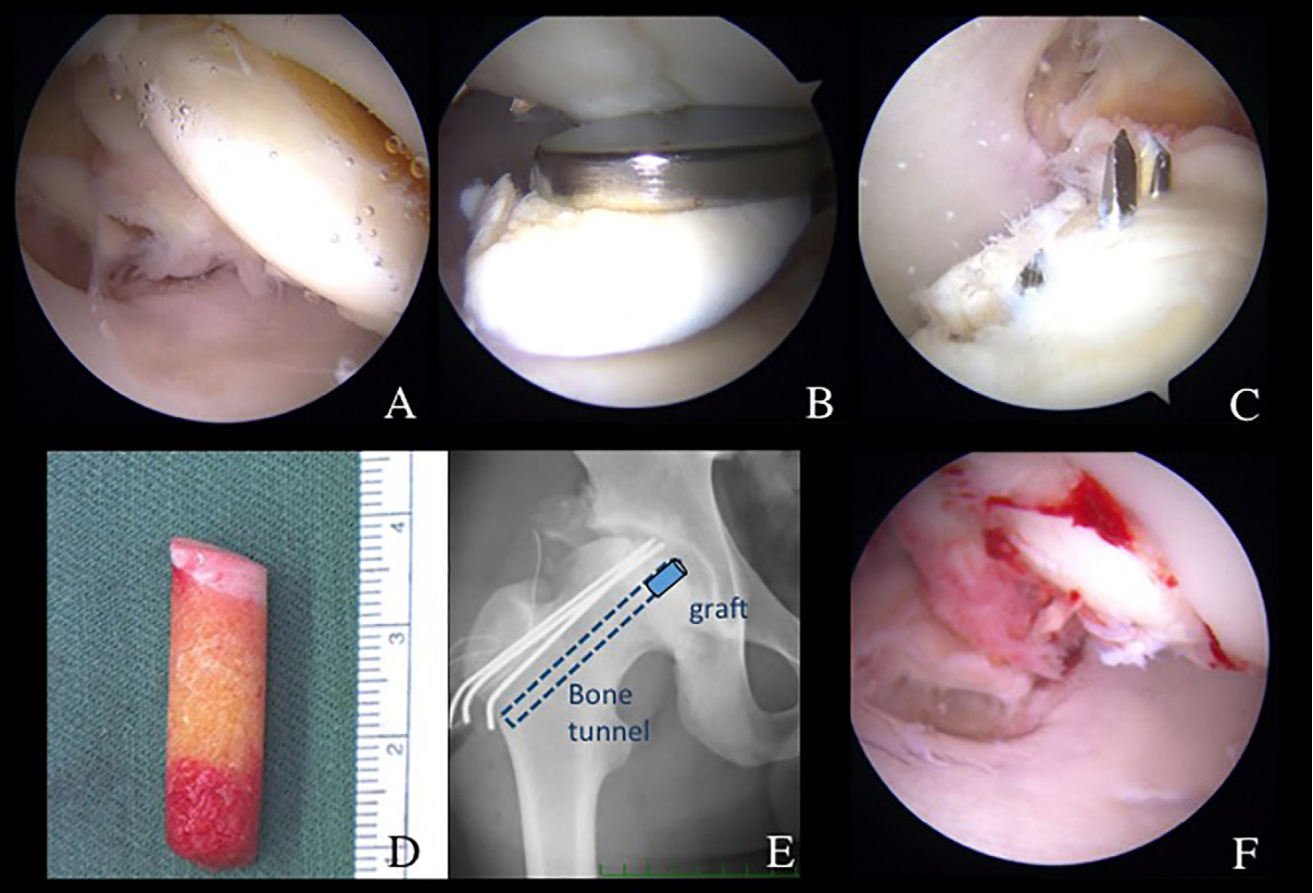
(A) Arthroscopic finding from ALP showing OCD lesion separated from the femoral head. (B) Viewing from MAP, OCD fragment was removed by forceps through ALP. (C) Viewing from PMAP, OCD was fixed by three 2 mm K-wires through CROSSTRAC guide. (D) Cylindrical osteochondral autograft harvested from femur. (E) Pelvic AP view showing retrograde pinning of K-wires and bone tunnel for OAT. (F) Viewing from ALP, OAT was delivered and fixed through the tunnel by using bone tunnel dilator from greater trochanter.

Since nonsurgical treatment failed, hip arthroscopy and retrograde osteochondral autograft transplantation were indicated. During the central compartment procedure, an anterosuperior acetabular labral tear (slightly degenerative flattened) was confirmed. Labral repair was performed with a suture anchor fixation (Figures 4A and 4B). After releasing traction, a dynamic assessment to evaluate impingement was performed confirming FAI cam impingement (Figure 4C). Cam osteoplasty was performed (Figure 4D). After reapplying traction, the OCD lesion (ICRS grade III) was observed in the weight-bearing area and perifoveal area of the femoral head (Figures 6A and 6B). Under fluoroscopy, a CROSSTRAC Hip Guide System (Smith & Nephew Endoscopy, Andover, MA) was introduced through the ALP (Figure 5). The aimer of CROSSTRAC GUIDE SYSTEM was placed at the center of a completely separated OCD lesion and a 2.4 mm guide-wire was drilled from greater trochanter with direct visualization from the MAP (Figure 5). Then, the aimer of CROSSTRAC GUIDE SYSTEM was placed at the partially separated OCD lesion and three 2.0 mm K-wires were introduced from the greater trochanter toward the articular surface of femoral head using CROSSTRAC GUIDE SYSTEM, performing a retrograde fixation with K-wires of the OCD lesion of the femoral head (Figure 6C and 6E). After releasing traction, a cylindrical autologous osteochondral graft (10 mm diameter) was harvested arthroscopically from the ipsilateral knee joint in a full extension of knee (Figure 2C). The superior lateral aspect of the intercondylar notch was used as the donor site. The 10-mm size Chizel (Smith & Nephew, Andover, MA) was introduced through the superolateral knee portal. It was driven into the bone to a depth of 25 mm. The harvester, containing the graft, was then removed by twisting the T-handle. For retrograde OAT procedure, a Core reamer (anterior cruciate ligament (ACL) drill guide system) was utilized to make a graft tunnel through the 2.4 mm ACL drill guide wire from greater trochanter to reach out to the articular surface of the femoral head (Figure 6F). The autologous OATS was delivered through the tunnel by using bone tunnel dilator from greater trochanter (Figure 6G).

**Figure 7.**
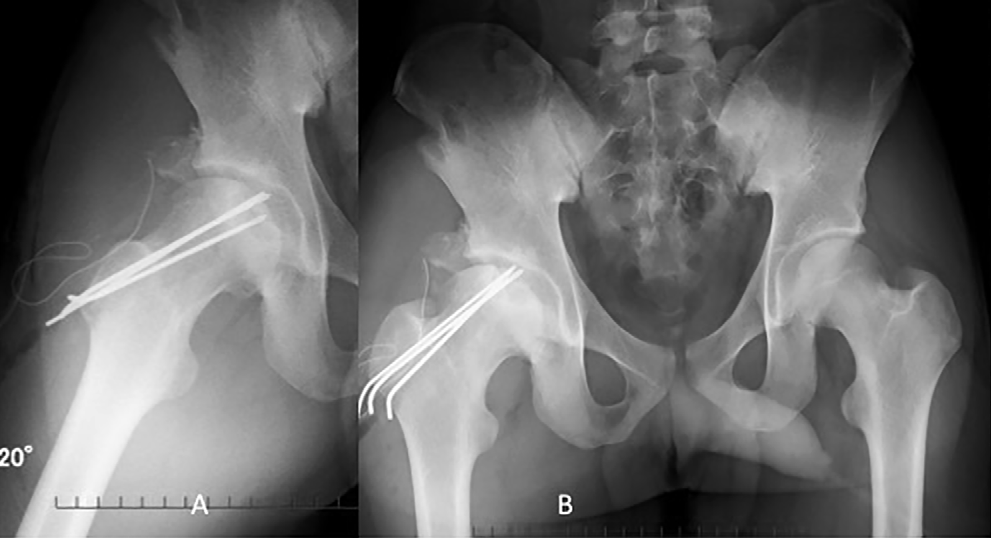
Postoperative anteroposterior (AP) pelvis and Dunn view confirming good healing after OAT treatment.

**Figure 8.**
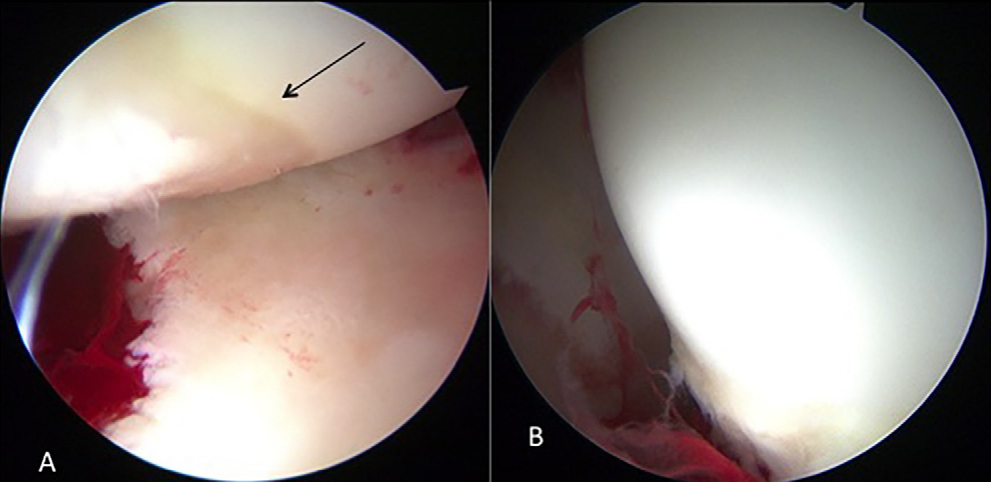
(A) Second look showing good healing of OAT and (B) fragment fixation at OCD lesion of the femoral head.

Postoperative X-ray showed good healing of OAT and retrograde fixation at OCD lesion of the femoral head (Figure 7). Since the patient required the removal of K-wires, second-look hip arthroscopy at 12 months was performed demonstrating good healing of the OCD lesion of the femoral head (Figure 8). Modified Harris hip score (MHHS) improved from 69.8 preoperatively to 100 at 14 months after surgery. Non-arthritis hip score improved from 88.7 to 100 at 14 months after surgery.

### Postoperative rehabilitation

Postoperative rehabilitation was used for both antegrade and retrograde OAT procedures. The patients were placed in a brace (Philippon brace, Bledsoe, Dallas, TX), for three weeks, to protect the hip and limit abduction and rotation. Gentle passive range of motion (ROM) exercise was initiated during the first week, under the supervision of a physiotherapist. The patient remained non-weight bearing, during the first four weeks. Active hip flexion was limited, during phase I (the first four weeks), to minimize the risk of hip flexor inflammation. From week four to five, weight bearing was gradually increased and the patient was progressed to full weight bearing at eight weeks after surgery. The patients were allowed to start swimming and do stationary bike with resistance at four months after surgery. Endurance strengthening began at 24 weeks after surgery; only after range of motion was maximized and after good stability in gait and movement was observed. Throughout this phase, there was no low impact aerobic conditioning.

## Discussion

In this study, we demonstrated two cases with OCD lesions of femoral head treated with an arthroscopic antegrade and retrograde OAT technique for treating OCD lesions in the hip.

In this article, the first case was associated with acetabular labral tear and the second case was associated with LCPD, acetabular labral tear, and hip dysplasia. There are several studies looking at OCD lesions with LCPD. Rowe et al. have shown that seven of 363 hips (about 2%) with LCPD have concomitant OCD lesions [12]. Steenbrugge and Macnicol reported four cases with OCD lesions of the femoral head diagnosed about four years following the occurrence of LCPD [4]. We think OCD lesion of femoral head may sequel to other hip pathologies including labral tears and/or LCPD.

Clohisy et al. described good clinical outcomes of residual Perthes like deformity and hip dysplasia treated with open surgical dislocation and concomitant periacetabular osteotomy [13]. Several studies have shown that shelf acetabuloplasty is also useful for treating Perthes like deformity with concurrent hip dysplasia [14, 15]. Recently, we developed a new endoscopic technique of shelf acetabuloplasty for treating hip dysplasia as well as intra-articular pathologies [16]. Thus, we utilized endoscopic shelf procedure concomitantly with arthroscopic OAT procedures.

There are several case reports of patients with osteochondral lesions of the femoral head undergoing OAT procedure. Nam et al. reported two cases of osteochondral lesions of the femoral head who had undergone OAT procedure combined with osteochondral fragment fixation after traumatic anterior dislocation of the hip joint. They demonstrated good clinical outcomes and graft incorporation showed by the MRI study [17]. Girard et al. demonstrated the surgical technique and clinical outcomes following OAT after treating 10 patients with OCD lesions of the femoral head. They revealed excellent well-healed incorporation of autologous graft in all radiographs [18]. Gagala et al. also reported good result following fragment fixation combined with OAT for treating osteochondral defect after posterior fracture dislocation of the hip joint. They proposed good clinical outcomes and graft congruity confirmed by MRI [19]. All those case reports described require an open surgical dislocation with a trochanteric osteotomy to assess and treat the osteochondral lesion. Contrary to arthroscopic procedures, open approaches could have higher rates of complications, such as, non-union of the greater trochanter and slower recovery [20].

Hip arthroscopy is less invasive and promising tool for assessing and treating hip intra-articular pathologies. Matsuda and Safran demonstrated good clinical outcomes after arthroscopic internal fixation of an OCD lesion in the femoral head using metal headless compression screws [21]. Arthroscopic OAT would be more advantageous if OCD lesion is irreparable and degenerative. Kubo et al. described a new surgical technique of arthroscopic antegrade OAT procedure for treating OCD at the anterosuperior aspect of the femoral head [8]. Cetinkaya et al. also described a surgical technique with arthroscopic retrograde OAT procedures for treating OCD lesion at the central weight-bearing area of the femoral head [9].

### Indication and contraindication

The indication of antegrade OAT technique is an OCD lesion (ICRS classification grades III and IV) at the anterior-superior or anterior-lateral aspect of the femoral head [22]. The indication of retrograde OAT technique is OCD lesion at center and/or posterior aspect of the femoral head. Contra-indication of these techniques are an infection surrounding hip joint and osteoarthritis (Table 1).

**Table 1.** Indication and contra-indication of OAT technique.

	Indication	Contraindication
Antegrade OAT	Osteochondral lesion grade III-IV (ICRS classification) located in the anterosuperior or anterior lateral aspect of the fermoal head.	Infection osteoarthritis
Retrograde OAT	Osteochondral lesion grade III-IV (ICRS classification) located in the center or posterior aspect of the femoral head.	

### Advantage and disadvantage

Arthroscopic OAT to treat OCD lesion of the femoral head has several advantages and some disadvantages. The advantages are that this procedure is minimally invasive and it has the potential for early rehabilitation and quicker recovery. Concurrent lesions can be readily assessed and treated arthroscopically. Surgeons are able to assess impingement issues and other intra-articular pathologies, such as, acetabular labral tears, ligamentous tearing, and cartilage delamination since OCD of the femoral head is often associated with other pathologies. However, considerable disadvantages are that this procedure is meticulous and technically demanding. The targeting of retrograde drilling is also is difficult despite the use of CROSSTRAC system. There is the possibility of donor-site morbidity, such as, donor-site pain in the knee joint.

### Conclusion

Hip arthroscopy plays a crucial therapeutic and diagnostic tool for the treatment of several hip pathologies including: labrum tears, cartilage damage and impingement. These reported techniques add a potential role of hip arthroscopy for the treatment of OCD.

## Conflict of interest

The authors declare no conflict of interest in relation with this paper.
